# Bisphenol A causes reproductive toxicity, decreases *dnmt1* transcription, and reduces global DNA methylation in breeding zebrafish *(Danio rerio)*

**DOI:** 10.1080/15592294.2016.1182272

**Published:** 2016-04-27

**Authors:** L. V. Laing, J. Viana, E. L. Dempster, M. Trznadel, L. A. Trunkfield, T. M. Uren Webster, R. van Aerle, G. C. Paull, R. J. Wilson, J. Mill, E. M. Santos

**Affiliations:** a Biosciences, College of Life & Environmental Sciences, Geoffrey Pope Building, University of Exeter, Exeter, EX4 4QD, United Kingdom; b University of Exeter Medical School, RILD building, University of Exeter, Exeter, EX2 5DW, United Kingdom; c Centre for Environment, Fisheries and Aquaculture Science (Cefas), Barrack Road, The Nothe, Weymouth, Dorset, DT4 8UB, United Kingdom; d Institute of Psychiatry, Psychology & Neuroscience (IoPPN), King's College London, Denmark Hill, London, SE5 8AF, UK

**Keywords:** Aquatic, endocrine, methylation, plasticizers, teleost, vertebrate, waste

## Abstract

Bisphenol A (BPA) is a commercially important high production chemical widely used in epoxy resins and polycarbonate plastics, and is ubiquitous in the environment. Previous studies demonstrated that BPA activates estrogenic signaling pathways associated with adverse effects on reproduction in vertebrates and that exposure can induce epigenetic changes. We aimed to investigate the reproductive effects of BPA in a fish model and to document its mechanisms of toxicity. We exposed breeding groups of zebrafish (*Danio rerio*) to 0.01, 0.1, and 1 mg/L BPA for 15 d. We observed a significant increase in egg production, together with a reduced rate of fertilization in fish exposed to 1 mg/L BPA, associated with significant alterations in the transcription of genes involved in reproductive function and epigenetic processes in both liver and gonad tissue at concentrations representing hotspots of environmental contamination (0.1 mg/L) and above. Of note, we observed reduced expression of DNA methyltransferase 1 (*dnmt1*) at environmentally relevant concentrations of BPA, along with a significant reduction in global DNA methylation, in testes and ovaries following exposure to 1 mg/L BPA. Our findings demonstrate that BPA disrupts reproductive processes in zebrafish, likely via estrogenic mechanisms, and that environmentally relevant concentrations of BPA are associated with altered transcription of key enzymes involved in DNA methylation maintenance. These findings provide evidence of the mechanisms of action of BPA in a model vertebrate and advocate for its reduction in the environment.

## Abbreviations

E2: 17β-estradiol
5Fc: 5-formylcytosine
5hmC: 5-hydroxymethylcytosine
*ar*: androgen receptor
*amh*: anti-Müllerian hormone
*cyp19a1a*: aromatase
BPA: bisphenol A
*dnmt3*: DNA (cytosine-5)-methyltransferase 3
*dnmt1*: DNA methyltransferase 1
*esr1*: estrogen receptor 1
*esr2a*: estrogen receptor 2a
*esr2b*: estrogen receptor 2b
ER: estrogen receptor
GSI: gonadosomatic index
HSI: hepatosomatic index
*hdac1*: histone deacetylase 1
*hdac3*: histone deacetylase 3
*mecp2*: methyl CpG binding protein 2
*mbd2*: methyl-CpG-binding domain protein 2
*mbd3a*: methyl-CpG-binding domain protein 3a
5mC: methylcytosine
PCA: principal component analysis
*rpl8*: ribosomal protein L8
*vtg1*: vitellogenin 1

## Introduction

Bisphenol A (BPA) is a commercially important high production chemical widely used in the production of epoxy resins, utilized in food and beverage packaging, dental sealants, and as a monomer component of polycarbonate plastics.^1,2^ With over three million tons produced globally per annum, environmental exposure is common,^3^ and in the USA BPA was measurable in 75% of food products tested.^4^ Human exposure occurs predominantly via ingestion of contaminated food, caused by leaching of BPA from linings of canned goods and polycarbonate packaging. BPA has also been detected in drinking water at concentrations up to 15 ng/L.^5^ In addition, inhalation is thought to be a plausible secondary route of exposure,^3^ with BPA present in 86% of domestic dust samples at concentrations ranging from 0.2 to 17.6 µg/g.^6^ BPA has been detected in the urine of ∼95% of adults in the USA and Asia.^7,8^ It has also been measured in the serum of adult men and women ^9^ and in breast milk, fetal plasma, and placental tissue, raising concerns about human exposures during critical periods of development.^1,10^

BPA is moderately water soluble, entering the environment via direct discharge from BPA production and processing industries, wastewater treatment plants and leachate from landfill sites.^11^ Its presence is ubiquitous in the aquatic environment and surface water concentrations have been detected up to the low µg/L range, with peak concentrations reaching up to 21 µg/L.^12^ Concentrations in landfill leachate have been reported to reach up to 17,200 µg/L.^1^ Due to its ubiquitous nature, the potential for environmental exposure in wildlife populations, including fish, is very significant. Levels of BPA reported in fish vary; values of 1-11 ng BPA/g dry weight in muscle and 2-75 ng BPA/g dry weight in liver have been reported.^13^

BPA has been shown to act as an estrogen receptor (ER) agonist,^14,15^ able to bind to ERs, resulting in feminizing effects.^16,17^ A study using the human cell line HepG2, found that BPA strongly activated estrogen receptor 1 (ESR1; previously known as ERα) mediated responses, but did not activate ESR2 (previously known as ERβ), while in the cell line HeLa, BPA was found to activate both ESR1 and ESR2.^14^ In fish, BPA induced *esr1* expression in the livers of male fathead minnows *(Pimephales promelas)* exposed for 4 d to 10 μg BPA/L, consistent with an estrogenic mode-of-action.^18^ BPA has also been shown to alter the transcriptional profile of steroidogenic enzyme genes in a time-dependent manner, including aromatase (*cyp19a1a*), which is responsible for the irreversible conversion of androgens into estrogens and is a key regulator of estrogen synthesis in the gonads. This enzyme was significantly upregulated in both the ovary and testis of *Gobiocypris rarus* exposed to 15 µg/L BPA for 7d, followed by suppression after 35 d exposure.^19^

Adverse impacts on reproduction have been observed in several fish models. A multi-generational study in fathead minnow showed that BPA reduced gonadal growth in males and females, reduced hatching in F1 offspring of fish exposed to 640 µg/L and induced the estrogen regulated egg yolk protein, vitellogenin, a well established biomarker of xenoestrogen exposure, in the liver of male fish exposed to 640 and 1280 µg/L BPA.^20^ Further multigenerational studies have demonstrated the potential adverse effects associated with exposure to BPA.^21,22^ Exposure to BPA in guppies has been associated with reduced sperm quality,^23^ and the presence of necrotic cells in the seminiferous tubules of *Xiphophorus helleri* was also reported.^24^ Together, these studies demonstrate the potential reproductive consequences following exposure to relatively high concentrations of BPA in fish.

Evidence also exists supporting the involvement of BPA in the etiology of a range of human disease phenotypes including cardiovascular disease,^25^ altered behavior in children,^26^ prostate cancer^27^ and recurrent miscarriage.^28^ In addition to the well-established estrogenic mode-of-action, additional mechanisms have been proposed, including potential anti-androgenic activity.^29^ Low dose effects and non-monotonic dose response curves have been reported.^30,31^ More recently, increasing evidence suggests that BPA may alter the epigenetic regulation of gene expression; for example, altered DNA methylation patterns have been observed both globally (i.e., changes to the total genomic content of DNA methylation) and at the regulatory regions of specific genes (i.e., locus-specific) in mammals.^32-36^ In humans, exposure to BPA in the workplace has been associated with hypomethylation of LINE-1 in spermatozoa, a marker of global DNA methylation levels in the genome.^37^ Understanding the effects of BPA exposure on epigenetic processes, and how these alterations perturb expression of genes that are related to development and reproduction, are important to the evaluation of adverse effects associated with BPA exposure, both in humans and wildlife, particularly for exposures at environmentally relevant concentrations.

To date, few studies have investigated the potential for BPA to induce epigenetic and transcriptional changes in fish. A study in *Gobiocypris rarus* found BPA exposure to be associated with altered DNA methylation in the 5′ flanking region of *cyp19a1a* (aromatase), and the effects to be time-dependent.^19^ In addition, a significant decrease in the expression of DNA methyltransferase 1 (*dnmt1*) in ovarian tissue has been reported, with a significant decrease in global DNA methylation.^19^

Given the extensive use and ubiquity of BPA, it is important to understand the mechanisms mediating its toxic effects and the impacts these can have on both wild populations and human health. The present study aims to investigate the effects of BPA on reproduction in the zebrafish model and identify epigenetic and transcriptional changes associated with BPA exposure. We exposed breeding groups of zebrafish to BPA for 15 d to determine if reproduction was affected by the exposure. The concentrations tested included environmentally relevant concentrations found world-wide (0.01 mg/L) and at point sources (0.1 mg/L).^12,38^ The highest concentration tested (1 mg/L) has only been reported in landfill leachate and is unlikely to occur in surface waters, but it was included to enable a mechanistic analysis of BPA toxicity. We quantified the transcription of genes involved in epigenetic signaling and reproductive function, together with global and locus-specific DNA methylation in exposed fish.

## Results

### Water chemistry

The mean measured concentrations of BPA in the tank water were between 100 and 139% of the nominal concentrations for all treatments, and are presented in Supporting Information Table S1.

### Effects of BPA on morphometric parameters

The mean mass and length of male and female fish were 460.0 ± 0.008 mg and 36.5 ± 0.02 mm, and 480.6 ± 0.01 mg and 35.7 ± 0.03 mm, respectively. There were no significant differences in size or condition factor (mean 0.95 and 1.05 for males and females, respectively) between treatment groups.

No alterations in general feeding and swimming behavior were observed in any spawning group, with the exception of the mortality of one female in the 0.1 mg/L BPA treatment. The egg output calculations for that group were adjusted accordingly. Hepatosomatic index (HSI; the ratio of liver weight to body weight) in males was significantly increased in fish exposed to 1 mg/L BPA, but no effects of BPA were observed in females (Supporting Information Fig. S1). There were no significant differences in the gonadosomatic index (GSI; the ratio of gonad weight to body weight) of males or females as a result of the BPA exposure.

### Effects of bisphenol A on reproduction

**Figure 1.**
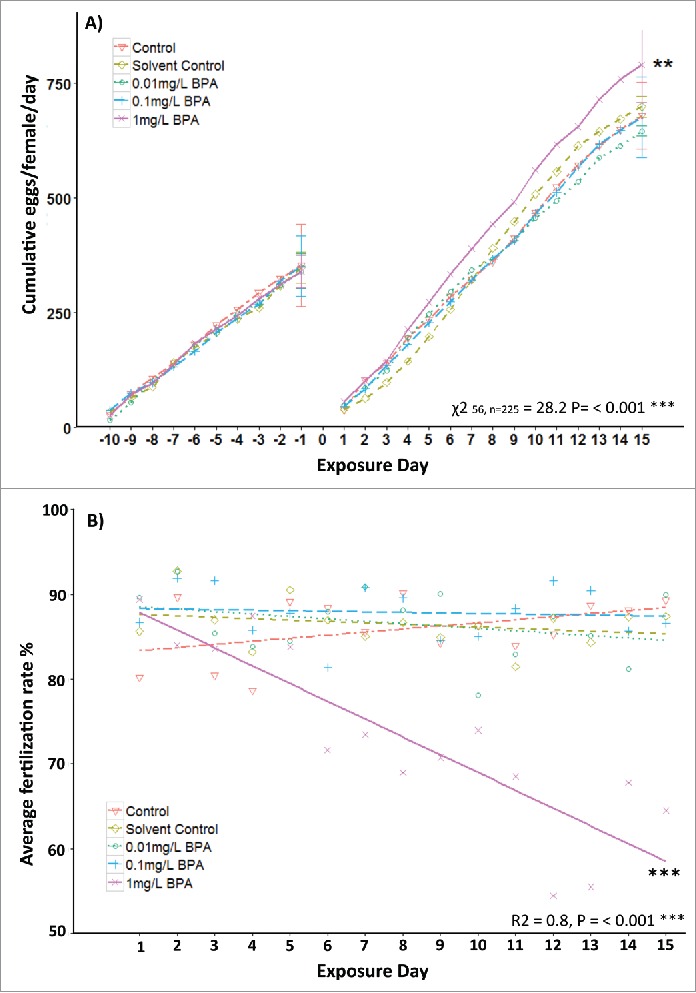
A) Cumulative number of eggs per female per day in breeding groups exposed to 0.01, 0.1, and 1 mg/L BPA. Data is presented for a 10 d pre-exposure followed by a 15 d chemical exposure periods (n = 3 replicate groups per treatment). Statistical comparisons were conducted in R (version 3.0.2), and the lme4 package was used to fit mixed effects linear models, followed by repeated measures ANOVA and Chi-squared Wald test to determine the effects of the exposure to BPA compared to the solvent control. B) Mean fertilization success (%) during the 15 d chemical exposure period (n = 3 replicate groups per treatment). Statistical analyses were conducted using R (version 3.0.2); the Regression coefficient (R^2^) was calculated using linear modeling. Asterisks indicate significant differences between treatment groups (**P < 0.01; ***P < 0.001).

During the 10 d pre-exposure period there were no differences in cumulative egg production between treatment groups (*P* = 0.098). During the exposure, groups treated with 1 mg/L BPA spawned a significantly greater number of eggs per female when compared to all other treatment groups (*P* ≤ 0.01); this increased egg production intensified throughout the exposure period (Fig. 1A). During the pre-exposure, fertilization success remained consistently high with no significant differences between groups and an overall mean fertilization rate of 85.6%. During the 15 d exposure, fertilization success in colonies exposed to 1 mg/L BPA significantly declined (*P* = 0.001; Fig. 1B). Additionally, for this treatment group, there was a significant negative correlation between the length of the exposure (number of days) and the average percentage of fertilization (R^2^ = 0.80; *P* ≤ 0.001), indicating that the effects of BPA on fertilization became progressively more pronounced over the exposure period.

### Effects of bisphenol A on gene transcription

**Figure 2.**
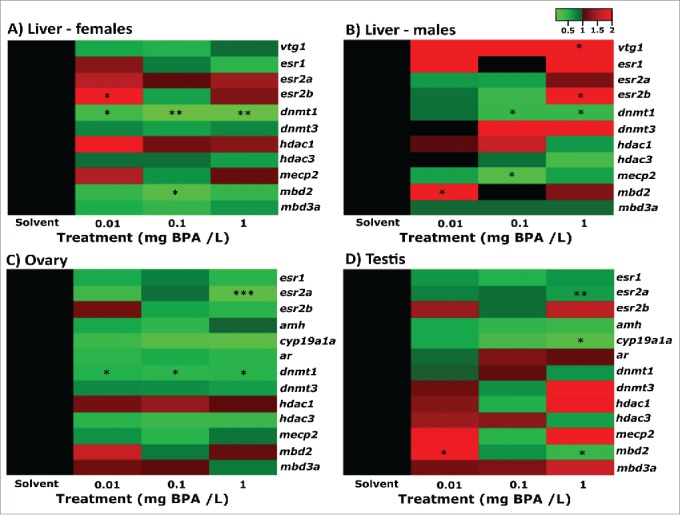
Transcript profiles for target genes in the livers of females (A) and males (B), and in the ovary (C) and testis (D) following exposure to 0.01, 0.1, and 1 mg/L BPA for 15 d. Data were collected for 6-8 fish per treatment, and data points classified as outliers (using the Chauvenet's criterion) and for which the expression was below the detection limit of the assay were excluded from analysis. Where amplification was detected in more than 70% of individuals, data are represented as fold-change relative to the expression in the solvent control group. Where amplification was detected in less than 70% of individuals, data are presented as the proportion of individuals for which the target genes were amplified. Asterisks represent significant differences between treatment groups compared to the solvent control group (*P < 0.05, **P < 0.01, ***P < 0.001).

Analysis of genes involved in reproductive processes in the liver revealed that *vtg1* and *esr2b* were significantly upregulated in males following exposure to 1 mg/L BPA when compared to the solvent control group (fold-change = 172.90, *P*=0.009 and fold-change = 5.40, P=0.014, respectively). In females, *esr2b* was significantly upregulated following exposure to 0.01 mg/L BPA (*P* = 0.044). For genes involved in epigenetic regulation, the most pronounced changes observed were for *dnmt1*, which was significantly downregulated in the livers of females exposed to 0.01 mg/L BPA (*P* = 0.040) and in both males and females exposed to 0.1 (males: *P* = 0.020; females: *P* = 0.005) and 1 mg/L BPA (males: *P* = 0.020; females: *P* = 0.005). In addition, changes were also observed for histone deacetylase 3 (*hdac3*), methyl-CpG-binding domain protein 2 (*mbd2*) and methyl CpG binding protein 2 (*mecp2*) in males, and for *mbd2* in females (Fig. 2A and B; Supporting Information Figs. S2 and 3).

**Figure 3.**
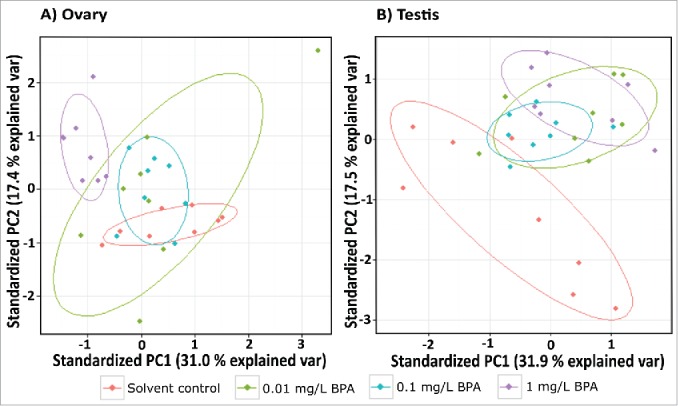
Principal components (PC) score plots showing the relative similarity of gonadal transcription profiles for zebrafish exposed to solvent, 0.01, 0.1, and 1 mg/L BPA for 15 d. A) Ovary. B) Testis. Points represent PC scores for individual fish along PCs 1 and 2. Circles represent a general characterization of the PC space occupied by each treatment group and were calculated using the prcomp package in R (version 3.0.2).

In the gonads, BPA exposure was also associated with significant changes in transcription for genes involved in reproductive function and on epigenetic pathways (Figs. 2, 3). Principal component analysis (PCA) for the testis indicated clear separations between the transcription profiles of fish exposed to the solvent control and fish exposed 1 mg/L BPA, based on the data for all genes quantified (Fig. 3). For ovaries, changes were more pronounced and PCA revealed a separation between fish exposed to 0.1 and 1 mg/L BPA and the solvent control (Fig. 3).

In the testis, the transcript encoding *esr2a* and *cyp19a1a* were significantly downregulated in response to 1 mg/L BPA (*P* = 0.002 and 0.018, respectively; Fig. 2; Supporting Information Fig. S4). There was also a significant association between the concentration of BPA and the level of transcription for *cyp19a1a* (*P* = 0.025; Supporting Information Table S4), which decreased with increasing concentrations of BPA. In addition, for anti-Müllerian hormone (*amh*), BPA affected gene transcription (*P* ≤ 0.05) and a decreasing trend across all concentrations was observed, but this was not statistically significant (*P* = 0.094; Supporting Information Fig. S4). Similarly to the testis, in the ovaries of exposed females, the transcript encoding *esr2a* was significantly downregulated following exposure to 1 mg/L BPA (*P* ≤ 0.001). In addition, there were similar (but non-significant) trends for other genes involved in reproductive function including *esr1* and *ar*, which appeared to decrease with increasing exposure concentrations (Fig. 2; Supporting Information Fig. S5).

As in the liver, *dnmt1* was significantly downregulated in ovaries following exposure to all three BPA concentrations tested (*P* = 0.032, 0.032, 0.032, respectively). Although no significant group-wise changes in *dnmt1* transcription were observed in the testis (Fig. 2; Supporting Information Fig. S4), the expression of *dnmt1* in the testis was associated with BPA exposure concentration (R^2^ = 0.110; *P* = 0.046; Supporting Information Table S4). In addition, changes in *mbd2* transcription were observed in the testis, with a significant increase in transcription measured in males exposed to 0.01 mg/L BPA (*P* = 0.020), but reduced expression in males exposed to 1 mg/L BPA (*P* = 0.030; Fig. 2; Supporting Information Fig. S4).

### Effects of bisphenol A on global DNA methylation

**Figure 4.**
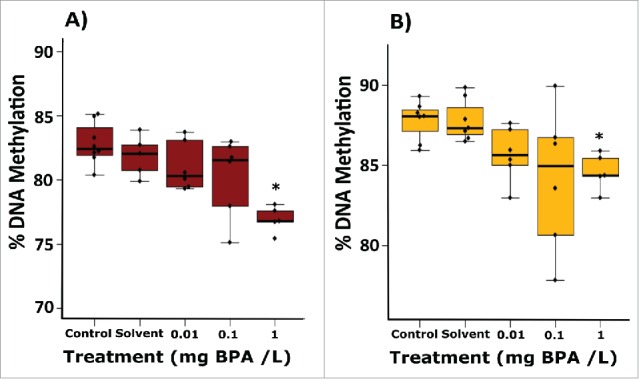
Global DNA methylation profiles in the gonads of adult zebrafish following exposure to 0.01, 0.1, and 1 mg/L BPA. Graphs present the percentage of global DNA methylation in ovaries (A) and testis (B). Data are presented as boxplots (n = 6-8 for each group). Asterisks indicate significant differences compared to the solvent control (*P < 0.05, **P < 0.01, ***P < 0.001).

Analysis of global DNA methylation in the gonads revealed significant decreases in the proportion of global methylation following exposure to 1 mg/L BPA in both males (by 3.2%; *P* = 0.029; Fig. 4A) and females (by 4.9%; *P* = 0.041; Fig. 4B).

### Effects of bisphenol A on gene-specific DNA methylation

**Figure 5.**
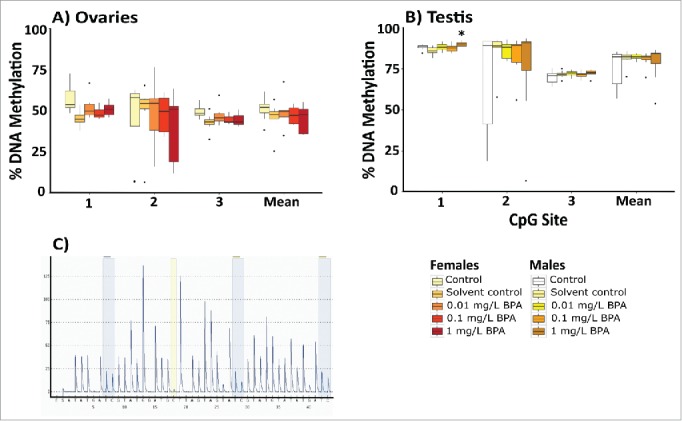
Gene specific DNA methylation profiles for three CpG sites in the promoter region of anti-Müllerian hormone (*amh*) in the ovaries (A) and testes (B) of adult zebrafish following exposure to 0.01, 0.1, and 1 mg/L BPA. C) Example pyrogram of three CpG sites in the 5′ flanking regions of the *amh* gene. Data are presented as boxplots (n = 6-8 for each group). Asterisks indicate significant differences compared to the solvent control (*P < 0.05, **P < 0.01, ***P < 0.001).

**Figure 6.**
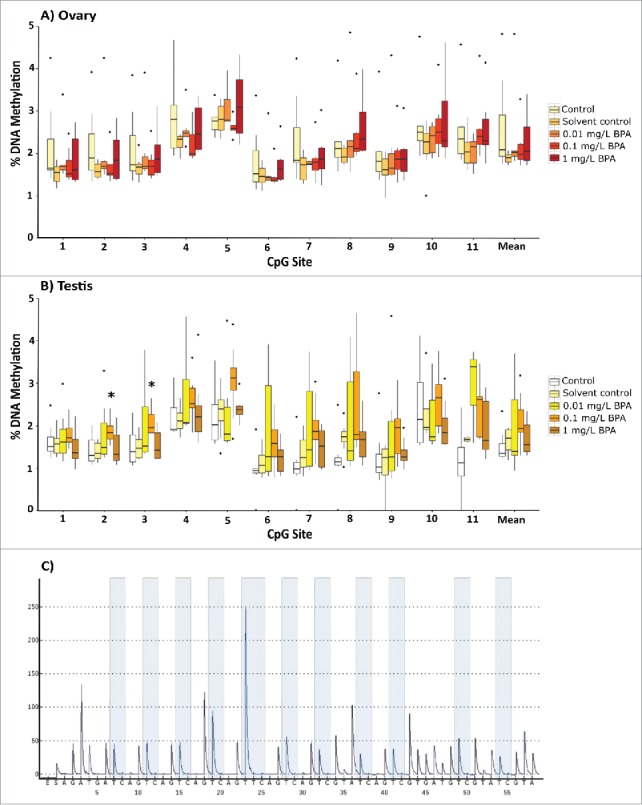
Gene-specific DNA methylation profiles for 11 CpG sites in the promoter region of DNA (cytosine-5)-methyltransferase 1 (*dnmt1*) in the ovaries (A) and testis (B) of adult zebrafish following exposure to 0.01, 0.1, and 1 mg/L BPA. C) Example pyrogram of 11 CpG sites in the 5′ flanking regions of the *dnmt1* gene. Data are presented as boxplots (n = 6-8 for each group). Asterisks indicate significant differences compared to the solvent control (*P < 0.05, **P < 0.01, ***P < 0.001).

**Figure 7.**
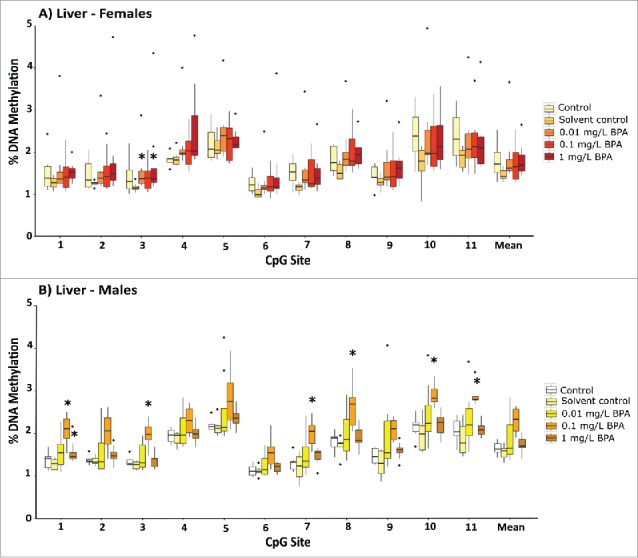
Gene specific DNA methylation profiles for 11 CpG sites in the promoter region of DNA (cytosine-5)-methyltransferase 1 (*dnmt1*) in the livers of female (A) and male (B) adult zebrafish following exposure to 0.01, 0.1, and 1 mg/L BPA. Data are presented as boxplots (n = 6-8 for each group). Asterisks indicate significant differences compared to the solvent control (*P < 0.05, **P < 0.01, ***P < 0.001).

Targeted DNA methylation profiling in the promoter region of *amh* revealed that exposure to 1 mg/L BPA caused a small but significant increase in methylation compared to the solvent control for the first of the three CpG sites assessed in the testes (*P* = 0.032; Fig. 5, see Supporting Information Fig. S6 for the position of this CpG site), with DNA methylation at this site being significantly correlated with BPA exposure concentration (R^2^ = 0.1625; *P* = 0.013). No differences in DNA methylation were seen for this region in ovaries from exposed female fish (Fig. 5). BPA was also not associated with altered DNA methylation at two CpG sites in the 5′ flanking region of the *esr1* gene in either the liver or gonads (Supporting Information Fig. S7). The analysis of 11 CpG sites across the promoter of *dnmt1* identified significant increases in DNA methylation for a number of sites in the liver (in both males and females) and the testes (males). Although group-wise comparisons of this region revealed no significant differences in the female ovaries (Figs. 6 and 7), *dnmt1* promoter methylation was significantly correlated with BPA exposure at various sites (positions 4, 5, 6 and 8; Supporting Information Table S4).

## Discussion

Exposure to BPA resulted in a consistent downregulation of *dnmt1* transcription in the ovary and in the liver of both males and females following exposure to BPA, including at environmentally relevant concentrations in females. In association with this, we found a reduction in global DNA methylation, probably due to the decrease in *dnmt1* expression. At the highest concentration tested, BPA caused reduced fertilization, potentially via estrogenic mechanisms. Together, our data provide evidence of the molecular mechanisms of action of BPA and the potential for it to cause adverse health impacts in vertebrates.

### Reproductive effects of BPA on adult zebrafish

We provide evidence that BPA exposure results in an impairment of reproductive function in breeding zebrafish. These effects included an increase in the number of eggs spawned and a decrease in fertilization success in groups exposed to 1 mg/L BPA. A number of mechanisms may contribute to the observed effect of BPA on reproduction, including stimulation of estrogen responsive processes via the interaction of BPA or its metabolites with estrogen signaling pathways, as previously reported for a range of organisms.^39–41^ We have investigated the effects of BPA on the expression of transcripts involved in reproductive function and known to be directly or indirectly regulated by estrogens.

We found no evidence for significant alterations in the transcription of *esr1* or DNA methylation across the *esr1* promoter in the gonads and livers of both sexes, but a significant association between BPA concentration and decreased transcription was found for the livers of females, and a trend for reduced expression was also observed in the ovaries and testis, similar to that described previously.^31^ Disruption of ESR1 has been associated with alterations of spermatogenesis and subsequently infertility in mice,^42,43^ therefore suggesting that the apparent decrease in *esr1* transcript in the testis may contribute toward the observed decline in fertilization success at this concentration.

BPA was found to downregulate *esr2a* in both ovaries and testes, but not in the liver. Similarly, a decrease in *esr2a* transcription was reported in ovaries of *Gobiocypris rarus* exposed to 0.05 mg/L BPA for 35, d and was associated with disruption of oogenesis and the occurrence of atretic follicles.^31^ These findings concur with previous studies reporting that *esr2a* is more sensitive compared to *esr1*, to the natural estrogen, 17β-estradiol (E2).^41^ In contrast, BPA caused increased transcription of *esr2b* in the livers of males and females but not in the gonads, and, importantly, for females this effect was observed at the environmentally relevant concentration of 0.01 mg/L BPA. In parallel, BPA induced a significant increase in the transcription of the egg yolk protein, *vtg1*, and an increase in HSI in males, likely as a result of increased vitellogenin production in hepatocytes, indicating an association between the induction of *esr2b* in males and the induction of *vtg1*, as previously reported for fathead minnows.^44^ Together, these findings suggest that the effects of BPA on reproduction involve disruption of estrogen receptor signaling principally via *esr1* and *esr2b* in the liver, and *esr2a* in the gonads.

In addition to the disruption in estrogen receptor signaling, changes in sex steroid biosynthesis may have contributed to the observed disruption of reproduction in colonies exposed to 1 mg/L BPA. We found a significant decrease in *cyp19a1a* transcript in the testis of males exposed to 1 mg/L BPA, and a significant association between transcription and BPA exposure concentration. In ovaries, a decreasing trend was also observed. These findings suggest potential feedback mechanisms were activated to counteract the estrogen/androgen ratio imbalance caused by BPA, through reducing the irreversible conversion of testosterone into estrogens. Similar findings have recently been reported for the Chinese rare minnow (*Gobiocypris rarus*) following a long term exposure to BPA,^19^ and studies using the aromatase knockout (ArKO) mouse found ArKO males to have reduced fertility,^45^ demonstrating the critical role of aromatase in gametogenesis in males.

In the testis, a decrease in *amh* transcription was associated with increased BPA exposure concentrations. Similarly, in mammals, downregulation of AMH has been reported following exposure to BPA.^46,47^ Exposure to 1 mg/L BPA also caused significant DNA hypermethylation in the *amh* promoter in the testis (CpG 1), demonstrating that exposure to BPA caused epigenetic alterations at this specific gene locus. There was also a significant correlation between the level of methylation in CpG 1 and *amh* transcription, and with BPA exposure concentration. This suggests that epigenetic mechanisms may be playing a role in the observed decline in *amh* transcript in testis tissue, which in turn could have consequences for the functioning of the testis, resulting in de-masculinization.

Fertilization success decreased over time with the mean fertilization rate dropping from 89% on day 1 to 69% by day 15. These findings are consistent with those of Haubruge et al., who reported declines in sperm count of 40-75% in guppies exposed to 0.274 or 0.549 mg/L BPA.^23^ BPA exposure has been linked to male sexual dysfunction in humans, and urinary concentrations of BPA have been associated with declines in sperm concentration, motility, and morphology in men.^48^ The mechanism by which disruption of normal spermatogenesis takes place is hypothesized to be via disruption of the Sertoli cells, which are directly sensitive to xenobiotic chemicals, and whose functions are essential during spermatogenesis.^23^ Our data are in agreement with these findings and further document the importance of Sertoli cells as targets for BPA toxicity, by demonstrating its effects on *amh* and *cyp19a1a*, both expressed in these cells in the testis.

Changes in fertilization success may have occurred not only due to effects of BPA on spermatogenesis but also due to BPA-induced alterations in egg quality. Females exposed to 1 mg/L BPA produced an increased number of eggs, but these eggs may have lacked the quality required for fertilization success and embryo survival. Many factors contribute to egg quality, of which the hormonal environment during oogenesis is a critical one.^49^ The observed changes in the expression of estrogen receptors and the trends observed for *cyp19a1a* in females indicate a disruption of the estrogen/androgen balance within ovaries and consequent alterations in sex steroid signaling pathways, putatively leading to alterations in oogenesis and oocyte quality. This hypothesis is supported by previous studies in which BPA was shown to affect oogenesis.^50^ In addition, a study in pregnant mice exposed to BPA found gross abnormalities in the meiotic prophase of oogenesis, including synaptic defects, which were suggested to occur via Esr2 (ERβ) signaling.^51^ Interestingly, in the present study, changes were also observed in the expression of an ERβ subtype (*esr2a*) in the gonads of both sexes, suggesting similar mechanisms could be occurring.

### Effects of BPA on epigenetic regulation

There is now strong evidence demonstrating that BPA has the potential to induce changes in DNA methylation at both gene-specific and genome-wide levels in exposed organisms^32,33^; however, this has rarely been studied in fish.

In our study, we found a significant decrease in the expression of the DNA methylation maintenance enzyme, *dnmt1*, for all three BPA concentrations tested in ovaries of females—including at environmentally relevant concentrations—and the DNA methylation pattern in the promoter region of the *dnmt1* gene was found to be significantly associated with BPA exposure concentrations for four CpG sites. The expression of *dnmt1* is known to be associated with changes in global DNA methylation, and inactivation of *dnmt1* has been shown to cause global demethylation of the genome.^52^ In this regard, it was interesting that global DNA methylation levels were significantly decreased in ovarian tissue of fish exposed to 1 mg/L BPA, potentially as a consequence of the suppression in *dnmt1* transcription. In contrast, previous studies in *Gobiocypris* *rarus*, have reported global DNA hypermethylation in ovaries exposed to 0.015 mg/L BPA for 35, d^19^ suggesting these epigenetic effects may be concentration- and time-dependent, and potentially vary across vertebrate species. Importantly, *dnmt1* is reported to be an important maternal transcript involved in the regulation of DNA methylation during the first stages of embryo development, particularly prior to the zygote genome activation.^53,54^ Therefore, the significant decrease in the expression of *dnmt1* observed in ovaries of females exposed to all three concentrations of BPA could have potential consequences for the appropriate development of offspring, in addition to influencing the level of DNA methylation in the ovary of exposed females.

For males, *dnmt1* transcription was also negatively associated with BPA exposure concentrations and a significant hypermethylation of two CpG sites in the promoter region of the *dnmt1* gene in fish exposed to 0.1 mg/L BPA was observed. In addition, we measured a significant decrease in global DNA methylation in the testis of fish exposed to 1 mg/L BPA, suggesting that the BPA-induced reduction in global methylation is likely to be functionally linked to the decrease in *dnmt1* transcription. These data align with the reported hypomethylation of sperm associated with the presence of BPA in urine, in a study of male factory workers in China.^37^ There is evidence to suggest that DNA demethylation and methylation establishment events during early development are guided by the paternal DNA methylation program instructed by the sperm chromosomes.^55,56^ Therefore, it is plausible that changes to the global DNA methylation pattern in testes such as those reported for fish exposed to 1 mg/L BPA may have the potential to impact on the epigenetic reprogramming of embryos, with potential consequences for their subsequent development.

In the liver, we observed a significant decrease in *dnmt1* transcription in males and females, including at environmentally relevant concentrations, demonstrating the very significant impact of BPA on the expression of this key DNA methylation maintenance enzyme. In addition, we report significant hypermethylation of the promoter region of the *dnmt1* gene in both male and female livers. Based on the positive association between the expression of this gene and global DNA methylation, it is plausible that the suppression of *dnmt1* may impact on global methylation as seen in other tissues. However, this could not be measured in the liver due to technical limitations related to the amount of DNA obtained from this tissue. The fact that changes in the transcript and methylation profile for *dnmt1* occur at environmentally relevant concentrations highlights the potential for BPA to cause epigenetic effects in exposed organisms within current exposure scenarios.

It is important to note that global DNA methylation in this study, measured using the LUMA assay, provides only an estimate of the total DNA methylation across all areas of the genome and all cell types in a given tissue. Decreased *dnmt1* transcription may be causing demethylation of specific areas of the genome or within specific cell types, but this may not be detectable by a global measurement of DNA methylation, including all cell types simultaneously. This may explain why *dnmt1* transcription appears to be more sensitive to BPA exposure compared to global methylation measurements.

The transcript profile for *mbd2* was significantly altered following exposure to BPA in both male testis and female livers. *mbd2* belongs to a family of nuclear proteins capable of binding specifically to methylated DNA, and may also function to repress transcription from methylated gene promoters.^57^ We found also a significant decrease in *mecp2* transcription in male livers, a gene involved in transcriptional repression by associating with methylated CpG dinucleotides where it silences transcription by recruiting histone deacetylases, resulting in chromatin remodeling.^58^ In addition, in male livers a significant decrease in *hdac3* transcription was also observed. These findings suggest that BPA is not only interacting with the processes linked to DNA methylation, but also has the potential to disrupt processes linked to chromatin structure and potentially impact on gene function via these mechanisms.

Despite the advances in our understanding of the epigenetic and transcriptional consequences of BPA in a model vertebrate, there are some limitations to the methodologies used: the locus-specific DNA methylation measurements conducted were based on the sodium bisulphite treatment of genomic DNA and, therefore, cannot distinguish between DNA modifications such as 5-hydroxymethylcytosine (5hmC), 5-formylcytosine (5fC), and methylcytosine (5mC), which have unknown functional significance.^59^ In addition, we explored the methylation status of specific CpG positions, within the regulatory regions of select target genes, hypothesized to be targets of BPA toxicity. This hypothesis-driven approach was successful in identifying some important mechanisms of BPA toxicity but may have missed other interesting effects outside these targeted regions, as suggested by the effects of BPA on global methylation levels. In addition, the global and locus-specific methylation measurements reported in this study are single measurements of DNA methylation across multiple cellular populations and cell types within each tissue. Both the gonad and liver are comprised of a mixture of cell types, whose genomic methylation and transcriptional activity is unique to the function of each cell type. In the testis for example, a large percentage of the cellular composition is made up of sperm cells containing very little cytoplasm and limited transcriptional activity, and the genomic DNA of sperm cells is also known to be hypermethylated. In contrast, the ovary contains oocytes characterized by very large cytoplasm where transcripts are stored to support the initial stages of embryogenesis before embryonic genome activation. Therefore, the datasets collected for these tissues are strongly dependent on the cellular composition of the tissue. In future studies, a genome-wide approach to measure methylation and also histone modifications, as well as analysis of single cells or pure populations of cells, may help to further characterize the effects of BPA on epigenetic signaling pathways.

## Conclusions

Overall, we have found evidence that BPA caused significant disruption to reproduction in breeding zebrafish exposed to 1 mg/L BPA, likely via estrogenic mechanisms. The potential for BPA to cause disruption of reproduction shown here raises concerns for its toxicity when organisms are exposed to BPA in environments affected by other stressors, including other environmental endocrine disruptors with similar mechanistic pathways that may act additively to cause reproductive disruption. Importantly, BPA also caused significant alterations in the transcription of a number of genes involved in epigenetic regulation in both liver and gonad tissue, most notably on *dnmt1*, which occurred in conjunction with decreases in global DNA methylation. Of note, some changes were observed after exposure to environmentally relevant concentrations of BPA (0.01 mg/L), corresponding to current exposure scenarios for both humans and wildlife. These findings provide evidence of the adverse effects of BPA in a model vertebrate and advocate for BPA's replacement within consumer products and its reduction in the environment.

## Materials and methods

### Chemicals

All chemicals were obtained from Sigma-Aldrich, UK, unless stated otherwise.

### Fish husbandry

Wild type WIK strain adult zebrafish (originating from a stock population at the University of Exeter) were maintained according to the conditions reported in Paull et al.^60^ Prior to the start of the experiment, fish were randomly allocated into 18 breeding groups of 4 males and 4 females, kept in individual 15-L flow-through tanks and were allowed to breed naturally during an acclimation period of 7 d. After this period, colonies that failed to spawn consistently were removed prior to the start of the experiment. Mains tap water was filtered by reverse osmosis [Environmental Water Systems (UK) Ltd.] and reconstituted with Analar-grade mineral salts to standardized synthetic freshwater (final concentrations to give a conductivity of 300 mS: 122 mg/L CaCl_2_2H_2_O, 9.4 mg/L NaHCO_3_, 50 mg/L MgSO_4_7H_2_O, 2.5 mg/L KCl, 50 mg/L Tropic Marin Sea Salt), aerated, and heated to 28°C in a reservoir, before it was supplied to each aquarium using a flow-through system. Tanks were aerated and supplied with a flow rate of 48 L/day of water.^60^ Tank water was maintained at 28 ± 0.5°C and pH 7-7.5 and fish were maintained under a 12 h light:dark cycle, including dawn and dusk transition periods of 30 min. Fish were fed live *Artemia nauplii* once daily (ZM Premium Grade Artemia; ZM Ltd.) and TetraMin tropical flake food (Tetra; Melle, Germany) twice daily, to satiation.

### Exposures of breeding zebrafish to bisphenol A

The selected 15 groups that showed consistent breeding and behavioral patterns during the initial acclimation period were subjected to a 10 d pre-exposure period, followed by a 15 d exposure period. Reproductive data for the 10 d pre-exposure period were collected to ensure that all breeding groups were reproducing consistently and there were no differences between reproductive measurements for any of the breeding groups prior to the chemical exposure period. Three independent replicate breeding groups were assigned at random to each treatment. A flow-through system was used to dose the tanks for 15 d with three concentrations of BPA (0.01, 0.1, and 1 mg/L) using ethanol (0.0005%) as a solvent. An absolute control receiving water alone and a solvent control receiving the same concentration of ethanol as the chemical exposures were also included.

On day one of the exposure period, tanks were spiked with the appropriate amount of BPA to achieve the required exposure concentrations. Flow rates were monitored daily to ensure the chemical concentrations remained consistent and dosing stocks were replaced every day. Water samples from each tank were collected on days 5, 10, and 15 of the exposure, and were stored at −20°C until chemical analysis.

The effects of BPA on reproduction were determined by measuring the egg production and fertilization success of individual groups. Eggs were collected each morning approximately one hour post-fertilization (hpf), washed and transferred to petri dishes for analysis. The numbers of fertilized and unfertilized eggs were determined by visual inspection for each treatment using a dissection microscope (Motic DM143, Hong Kong).

On day 15 of the exposure period, all fish were sacrificed humanely using a lethal dose of benzocaine followed by destruction of the brain, in accordance with UK Home Office regulations. The wet weight and fork length were recorded, and the condition factor for each fish was calculated (k) = [weight (g) × 100]/[fork length (cm)]^3^. The gonads and livers were dissected and weighed, and the gonadosomatic index (GSI) = gonad weight (mg)/[total weight (mg)- gonad weight (mg)] × 100 and hepatosomatic index (HSI) = liver weight (mg)/[total weight (mg)- liver weight (mg)] × 100 were calculated. Gonads and livers were collected, snap frozen in liquid nitrogen and stored at −80°C until analysis for transcript profiling and DNA methylation.

### Transcript profiling

Transcript profiling of genes encoding epigenetic regulatory proteins and genes involved in reproductive function was conducted using real-time quantitative PCR (RT-QPCR) as previously described.^61^ Beacon Designer 3.0 software (Premier Biosoft International, Paulo Alto, CA) was used for designing primers for each target gene using zebrafish NCBI RefSeq sequences, and primers were purchased from MWG-Biotech (Ebersburg, Germany). Assays for each transcript were optimized and standard curves were generated as previously described.^61^ Primer specificity was confirmed by observation of a single amplification product of the expected melting temperature throughout the range of detection. The linear correlation (R^2^) between the mean Ct and the logarithm of the cDNA dilution was > 0.99 in each case, and efficiencies were between 1.86-2.24. The primer sequences, annealing temperatures, PCR product sizes and PCR efficiencies for each primer pair are shown in Supporting Information Table S2.

RNA and DNA were extracted together from the livers and gonads of 8 male and 8 female fish from each treatment group using the AllPrep DNA/RNA Micro Qiagen Kit (Qiagen, Hilden, Germany) according to the manufacturer's instructions, which allows for extraction of both RNA and DNA from the same tissue sample. NanoDrop ND-1000 Spectrophotometer (NanoDrop Technologies, Wilmington, USA) was used to assess RNA and DNA purity and concentration. RNA was treated with DNase I (Qiagen) to remove any potential DNA contamination. cDNA was synthesized from 2 µg of total RNA using random hexamers (MWG-Biotech, Ebersberg, Germany) and M-MLV reverse transcriptase (Promega, Madison, USA), according to manufacturer's instructions. cDNA was then diluted 1:2 and RT-QPCR was performed in duplicate using an iCycler iQ Real-time Detection System (Bio-Rad Laboratories, Hercules, CA) and SYBR Green chemistry, as previously described.^61^ On each plate, a template-minus negative control was run in duplicate to verify the absence of cDNA contamination. Efficiency-corrected relative expression levels were determined after normalization to a control gene, ribosomal protein l8 (*rpl8*), which has been shown to have stable expression in the livers and gonads following exposures to estrogens in another cyprinid fish species.^44,62^

### Bisulfite pyrosequencing

DNA sequence data for the promoter regions of *esr1, amh*, and *dnmt1* were obtained from Ensembl (release 83; Cunningham et al. 2015) ^63^ using the Biomart portal.^64^ Zebrafish *esr1* (ENSDARG00000004111) has 3 known transcripts [*esr1*-001 (3449 bp), *esr1*-201 (3502 bp) and *esr1*-202 (212 bp)] and 2 transcription start sites (TSSs). The *dnmt1* gene (ENSDARG00000030756) also has 2 TSSs and 3 transcripts [*dnmt1*-001 (4896 bp), *dnmt1*-201 (4893 bp) and *dnmt1*-202 (5031 bp)]. *amh* (ENSDARG00000014357) has one transcript (*amh*-001, 3243 bp) and one TSS (Supporting Information Fig. S6). Target sites within the promoter sequences were chosen based on their proximity to the TSSs and estrogen-responsive elements (EREs), identified using JASPAR,^65^ and the matrix models ESR1 (MA0112) and ESR2 (MA0258). PCR and bisulfite pyrosequencing assays were designed using the PyroMark Assay design software (Qiagen, Hilden, Germany). Pyrosequencing primers and their corresponding target sequences are shown in Supporting Information Table S3.

Template preparation and pyrosequencing was carried out as described by Tost and Gut (2007) ^66^ on bisulfite-treated DNA from 8 individual fish (gonads and livers) per treatment group. Briefly, genomic DNA (500ng) was treated with sodium bisulfite using the EZ-96 DNA Methylation-Gold Kit (Zymo Research, CA, USA) according to the manufacturers' standard protocol. Water negative controls were run in duplicate to verify the absence of DNA contamination. Bisulfite-PCR amplification was performed in duplicate using the primers and assay conditions provided in Supporting Information Table S3. Unmodified DNA samples were included during primer optimization to confirm primer specificity for bisulfite-modified DNA.

### Luminometric-based assay (LUMA) for global DNA methylation

The LUMA assay was performed as described by Karimi et al. (2006) using DNA extracted from gonad samples from 8 individual fish per treatment.^67^ Sufficient quantities of DNA were not available to perform the LUMA assay in liver samples; therefore, analysis of global DNA methylation were conducted only for gonad samples. Each DNA sample (250 ng) was digested in duplicate with HpaII and MspI, and data were normalized to the EcoRI peak to account for any technical differences between samples.^68^ Global DNA methylation values were calculated according to the formula [HpaII(G)/EcoRI(T)]/[MspI(G)/EcoRI(T)], where G and T refer to the peak heights for HpaII or MspI (methylation) and EcoRI (input DNA), respectively.

### Water chemistry

For analysis of the concentrations of BPA in the exposure water, methanol, acetonitrile and water, both HPLC and LC-MS grade, HiPerSolv CHROMANORM®, were purchased from VWR Int. One mL of each water sample was added to a glass vial and mixed with 1 mL of HPLC-grade acetonitrile. Before LC-MS/MS analysis, aliquots were vortexed and diluted in a mixture of acetonitrile and water (1:3 v/v). Analyses were performed using a Surveyor MS Pump Plus HPLC pump with an HTC PAL autosampler coupled to a TSQ Vantage triple quadrupole mass spectrometer equipped with heated electrospray (HESI II) source (ThermoFisher Scientific, Hemel Hempstead, UK). Chromatographic separation was achieved using a reversed-phase, 3 µm particle size, C18 Hypersil GOLD column 50 mm × 2.1 mm i.d. (Thermo Scientific, San Jose CA, USA). Analytes were separated using a linear gradient of water and methanol. The initial conditions for the gradient consisted of 10% methanol, which was increased to 100% in 4.5 min and maintained for 1 min before returning to the initial 10% methanol. The flow rate was 500 µL/min. The temperature of the autosampler was set at 8°C, and the column was kept at a room temperature. The HESI probe was operating in the negative mode and an ion-spray voltage of −4.0 kV was applied. The heated capillary temperature was set at 275°C and the vaporizer temperature was 60°C. Nitrogen was employed as sheath and auxiliary gas at a pressure of 30 and 5 arbitrary units, respectively. The argon CID gas was used at a pressure of 1.5 mTorr and the optimum collision energy (CE) for each transition was selected. Quantification of BPA was performed using two characteristic multiple reaction monitoring (MRM) transitions of precursor ion 227.1→ 212.1 (CE: 20 V) and 227.1 → 133.1 (CE: 28 V).

### Statistical analysis

Statistical analyses were carried out using R (version 3.0.2).^69^ Prior to analysis, data were tested for equal variance and for normality using the Shapiro–Wilk test. Proportional data and variables with non-Gaussian distributions or non-homogeneous variances were subjected to variance-stabilizing arcsine transformations or log transformations. Non-parametric statistics were used when transformations did not result in distributions meeting the assumptions for parametric tests. All graphs were plotted using untransformed data for ease of interpretation. For the mean fertilization rates, comparisons between treatments were performed using Kruskal-Wallis tests followed by the Wilcoxon signed rank test. The Regression coefficient (R^2^) was calculated using linear modeling for fertilization rates. Linear mixed effects models were generated using the lme4 package ^70^ in order to explore the effect of BPA concentration and length of exposure on egg numbers. Non-significant terms were removed from models; models were compared based on likelihood ratio testing to give the appropriate minimum adequate model. Model results were inspected to ensure residuals were normally distributed.

In order to determine the effects of BPA on the reproductive and molecular endpoints measured, statistical comparisons were performed between the solvent control and the groups exposed to BPA, and comparisons between the water control and the solvent control were also conducted to confirm that no significant differences occurred as a result of the presence of the solvent. Comparisons between treatments were performed using one-way analysis of variance (ANOVA) and Kruskal-Wallis tests. Where ANOVA analysis found a *P* ≤ 0.05, post-hoc testing was carried out using the pairwise multiple comparisons of means method with false discovery rate *P* value adjustment. Where the Kruskal-Wallis test was used, post-hoc testing was carried out using the Wilcoxon signed rank test accounting for repeated measures within the data sets. *P* values of ≤ 0.05 were considered to be significant. All data are presented as mean ± SEM.

For transcript profiles, data points classified as outliers (using Chauvenet's criterion) and data points for which the expression was below the assay detection limit were excluded from analysis. Where amplification was detected in more than 70% of individuals, data were represented as fold-change relative to the expression in the water control group and groups were then compared using one-way ANOVA and Kruskal-Wallis tests with post-hoc tests as described previously. Where amplification was detected in less than 70% of individuals, data were represented as the proportion of individuals for which the target genes were detected, and analysis was conducted using a binomial generalized linear model. In the gonadal data sets, PCA was also performed using the prcomp function to identify the main trends in gene expression.

In order to determine if there were associations between the methylation levels for specific loci in the promoter regions of genes of interest and their transcription, correlation analysis was conducted. Where data was normally distributed Pearson correlation was used, and where data did not meet the assumptions of parametric testing, Spearman correlation analysis was performed. Correlation analyses were also conducted to determine the relationship between global methylation and *dnmt1* transcription, as above. The relationship between BPA concentration and transcript expression or methylation was also determined using regression analysis, calculated using linear modeling.

All graphs were plotted using untransformed data for ease of interpretation, and were created using the R packages ggplot2,^71^ gplots,^72^ beeswarm,^73^ and ggbiplot.^74^

## Supplementary Material

KEPI_A_1182272_s02.docx
