# Supplementary material for: Bisphenol A causes reproductive toxicity, decreases dnmt1 transcription, and reduces global DNA methylation in breeding zebrafish (Danio rerio)
Source: Epigenetics. 2016 Apr 27;11(7):526–38. doi: 10.1080/15592294.2016.1182272 (PMC4939919; doi:10.1080/15592294.2016.1182272)
Supplement: KEPI_A_1182272_s02.docx [file kepi-11-07-1182272-s001.docx]

**SUPPORTING INFORMATION**

**Bisphenol A causes reproductive toxicity, decreases *dnmt1* transcription and reduces global DNA methylation in breeding zebrafish (*Danio rerio*).**

L. V. Laing^1^*, J. Viana^2^, E. L. Dempster^2^, M. Trznadel^1^, L. A. Trunkfield^1^, T. M. Uren Webster^1^, R. van Aerle^3^,G. C. Paull^1^, R. J. Wilson^1^, J. Mill^2,4^, E. M. Santos^1^*

^1^ Biosciences, College of Life & Environmental Sciences, Geoffrey Pope Building, University of Exeter, Exeter, EX4 4QD, United Kingdom

^2^ University of Exeter Medical School, RILD building, University of Exeter, Exeter, EX2 5DW, United Kingdom

^3^ Centre for Environment, Fisheries and Aquaculture Science (Cefas), Barrack Road, The Nothe, Weymouth, Dorset, DT4 8UB, United Kingdom

^4^ Institute of Psychiatry, Psychology & Neuroscience (IoPPN), King’s College London, Denmark Hill, London, SE5 8AF, UK.

* Corresponding authors

E-mail: [ll292@exeter.ac.uk](mailto:ll292@exeter.ac.uk), [e.santos@exeter.ac.uk](mailto:e.santos@exeter.ac.uk)

**
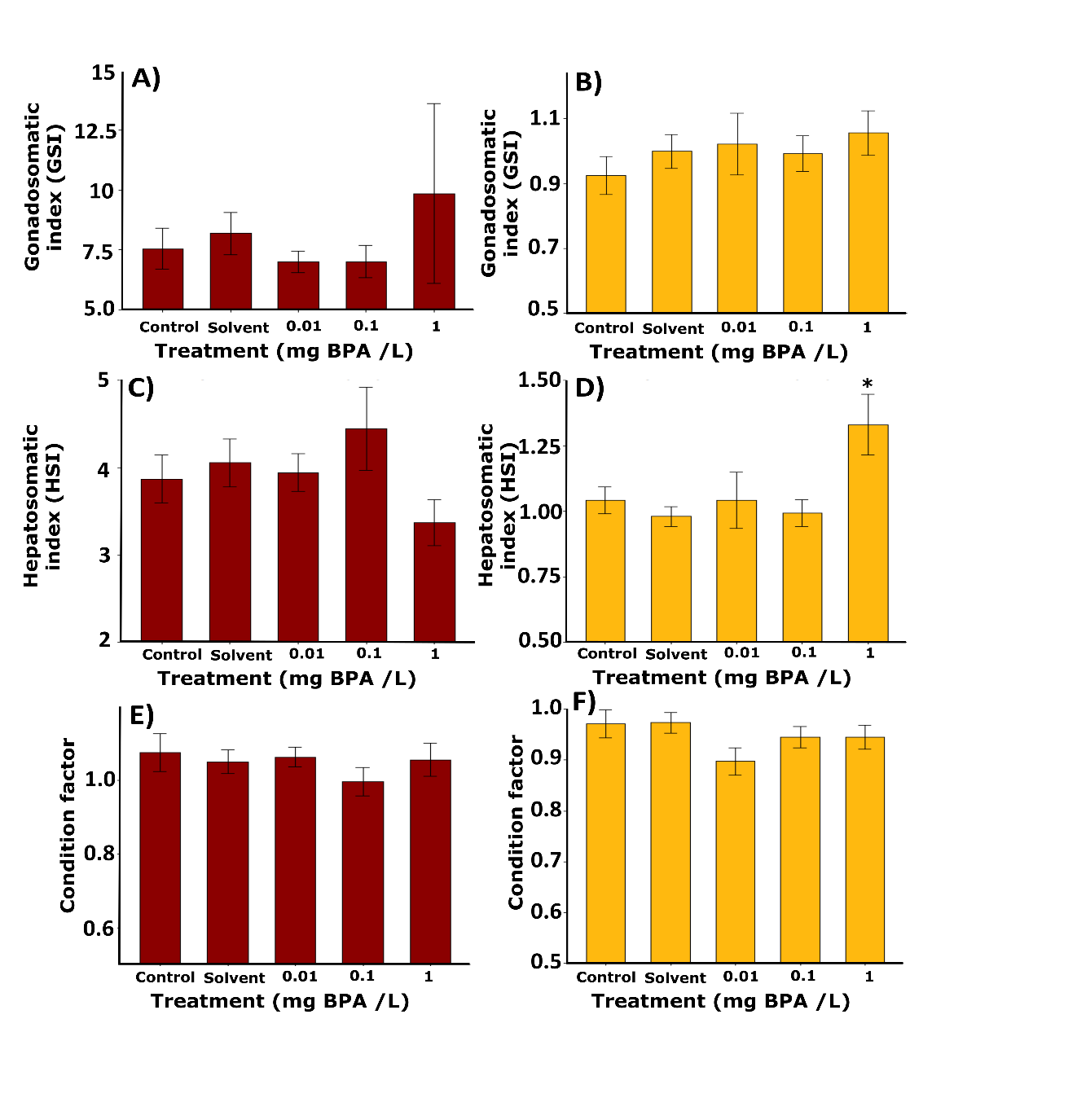
Supporting Information Figure S1.** Morphometric parameters for males and females exposed to 0.01, 0.1 and 1 mg/L BPA (n=12 individuals per treatment). Individual plots represent the gonadosomatic index for females **(A)** and males **(B),** hepatosomatic index for females **(C)** and males **(D),** and the mean condition factor for females **(D)** and males **(E).** Statistical comparisons were conducted using Kruskal-Wallis one-way ANOVA on ranks followed by the pairwise Wilcox test, in R (version 3.0.2). All data are presented as mean ±SEM. Asterisks indicate significant differences compared to the solvent treatment (*p<0.05).


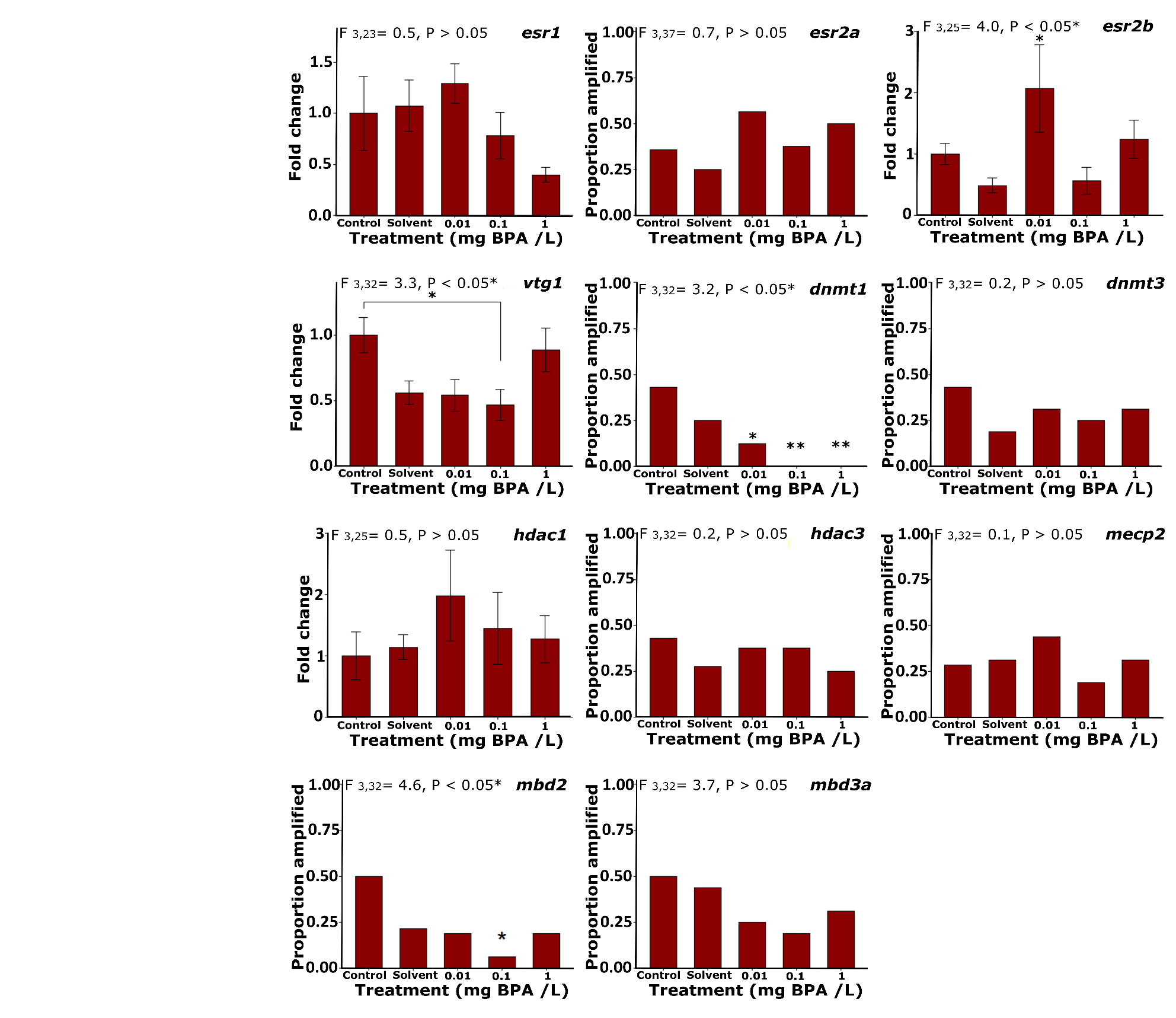


**Supporting Information Figure S2.** Transcript profiling of target genes in female livers following exposure to 0.01, 0.1 and 1mg/L BPA for 15 days. Data were collected for 6-8 fish per treatment, and data points classified as outliers (using Chauvenet’s criterion) and for which the expression was below the detection limit of the assay were excluded from analysis. Where amplification was detected in more than 70% individuals, data are represented as fold- change relative to the expression in the control group. Where amplification was detected in less than 70% individuals data are presented as the proportion of individuals for which the target genes were detected. Asterisks represent significant differences between treatment groups compared to the solvent control group (*P<0.05 **P<0.01 ***P<0.001).


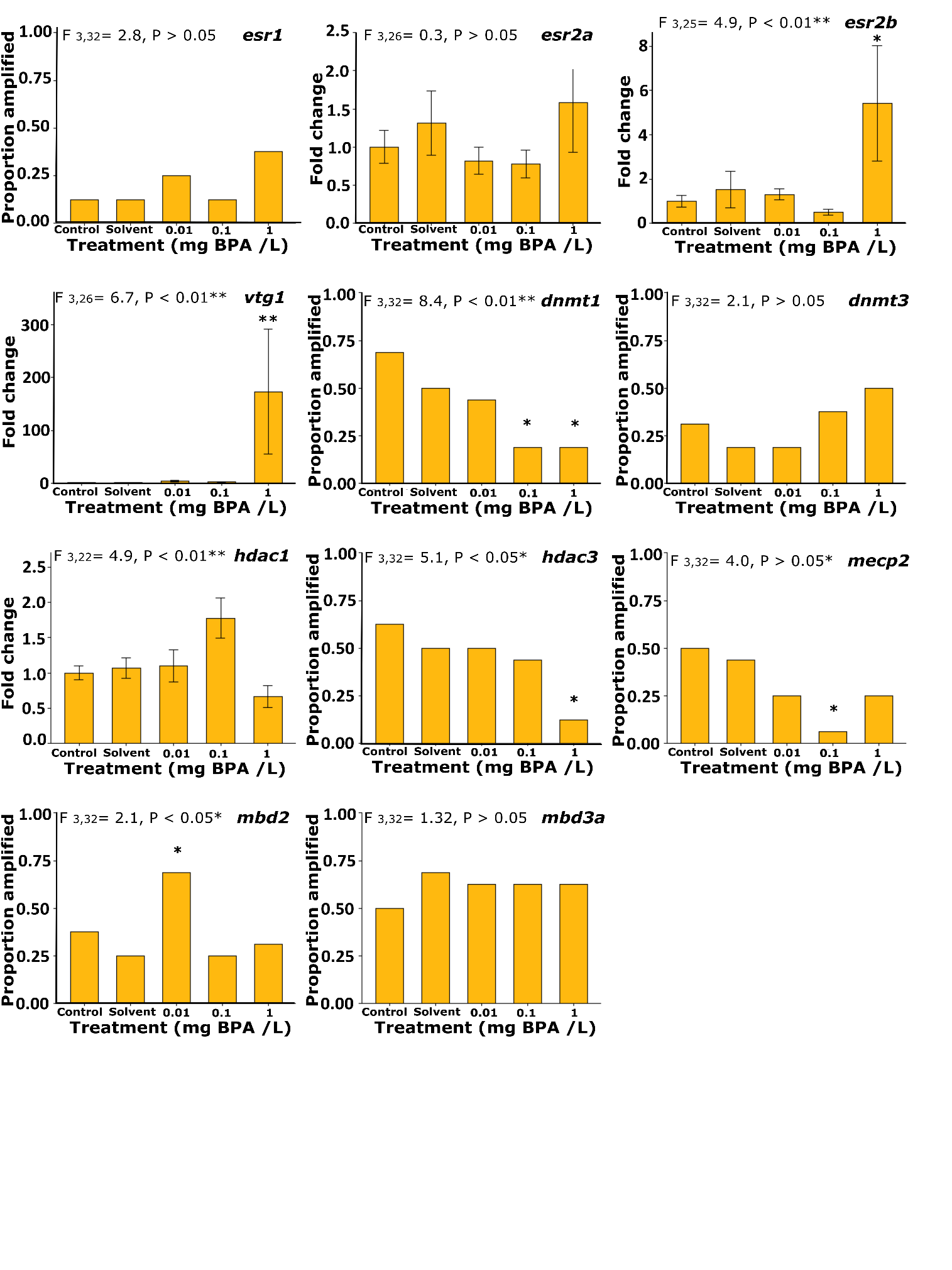


**Supporting Information Figure S3.** Transcript profiling of target genes in male livers following exposure to 0.01, 0.1 and 1mg/L BPA for 15 days. Data were collected for 6-8 fish per treatment, and data points classified as outliers (using Chauvenet’s criterion) and for which the expression was below the detection limit of the assay were excluded from analysis. Where amplification was detected in more than 70% individuals, data are represented as fold-change relative to the expression in the control group. Where amplification was detected in less than 70% individuals data are presented as the proportion of individuals for which the target genes were detected. Asterisks represent significant differences between treatment groups compared to the solvent control group (*P<0.05 **P<0.01 ***P<0.001).


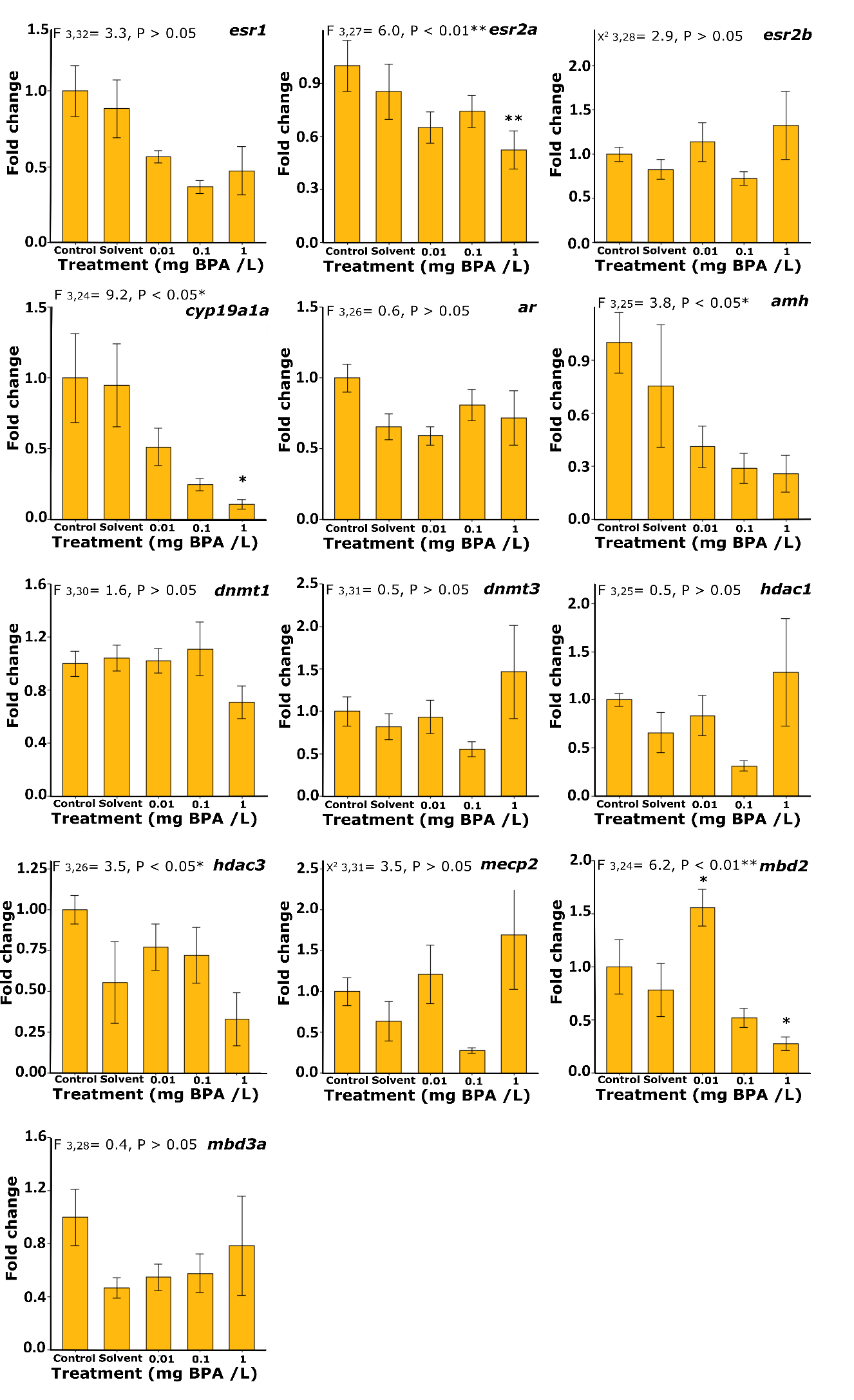


**Supporting Information Figure S4.** Transcript profiling of target genes in the testis following exposure to 0.01, 0.1 and 1mg/L BPA for 15 days. Data are presented as fold-change relative to the control group. Relative expression was calculated as a ratio of the efficiency corrected expression data for the target gene / efficiency corrected expression data for *rpl8*. For each treatment, data were obtained for 6–8 individual fish. Individual data points classified as outliers, and for which the expression was below the detection limit of the assay were excluded from the analysis using the Chauvenet’s criteria. Asterisks represent significant differences between treatment groups compared to the solvent control group (*P<0.05 **P<0.01 ***P<0.001).


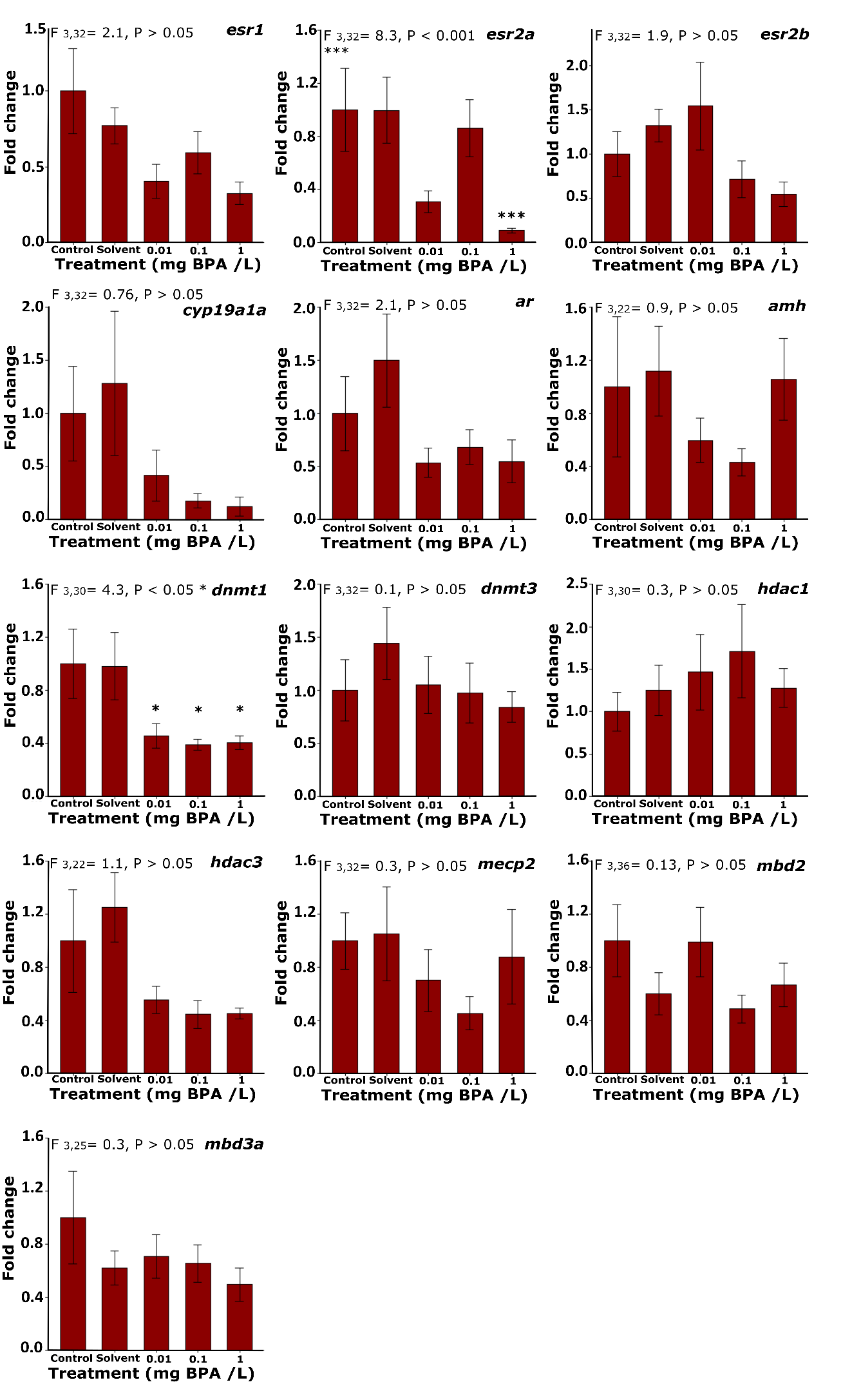


**Supporting Information Figure S5.** Transcript profiles of target genes in ovaries following exposure to 0.01, 0.1 and 1mg/L BPA for 15 days. Data are presented as fold-change relative to the control group. Relative expression was calculated as a ratio of the efficiency corrected expression data for the target gene / efficiency corrected expression data for *rpl8*. For each treatment, data were obtained for 6–8 individual fish. Individual data points classified as outliers, and for which the expression was below the detection limit of the assay were excluded from the analysis using the Chauvenet’s criteria. Asterisks represent significant differences between treatment groups compared to the solvent control group (*P<0.05 **P<0.01 ***P<0.001).

**
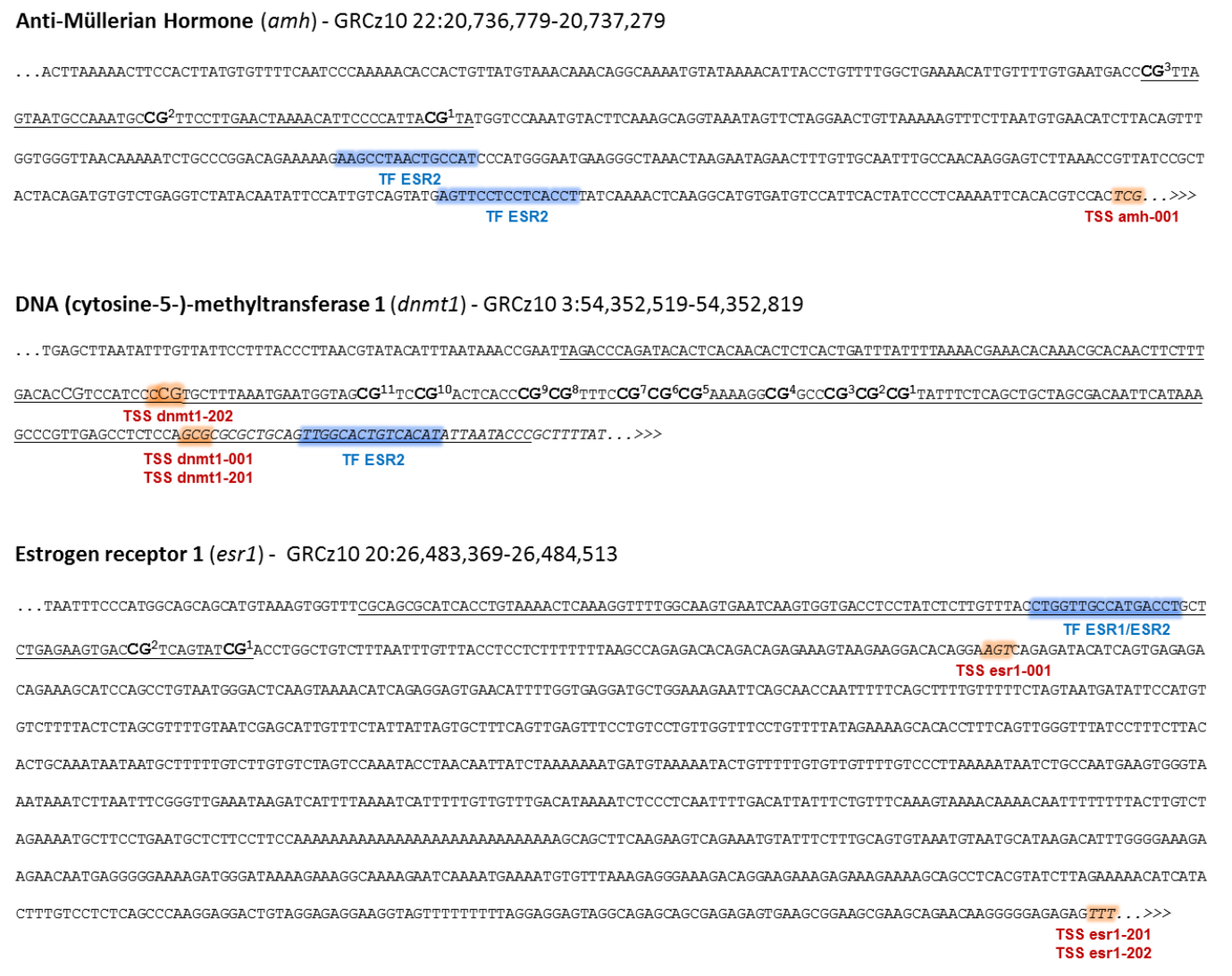
**

**Supporting Information Figure S6.** Promoter regions of *amh*, *dnmt1* and *esr1*, showing the location of the CpG sites (indicated in bold), the target sequences used for pyrosequencing (underlined) and putative EREs (highlighted in blue) in relation to the transcription start sites (TSSs; highlighted in red). The sequences shown were derived from Ensembl Zv9 (release 83; assembly GRCz10) and correspond to the following genomic positions: chr22:20,736,779-20,737,279 (*amh*), chr3:54,352,519-54,352,819 (*dnmt1*) and chr20:26,483,369-26,484,513 (*esr1*).

**
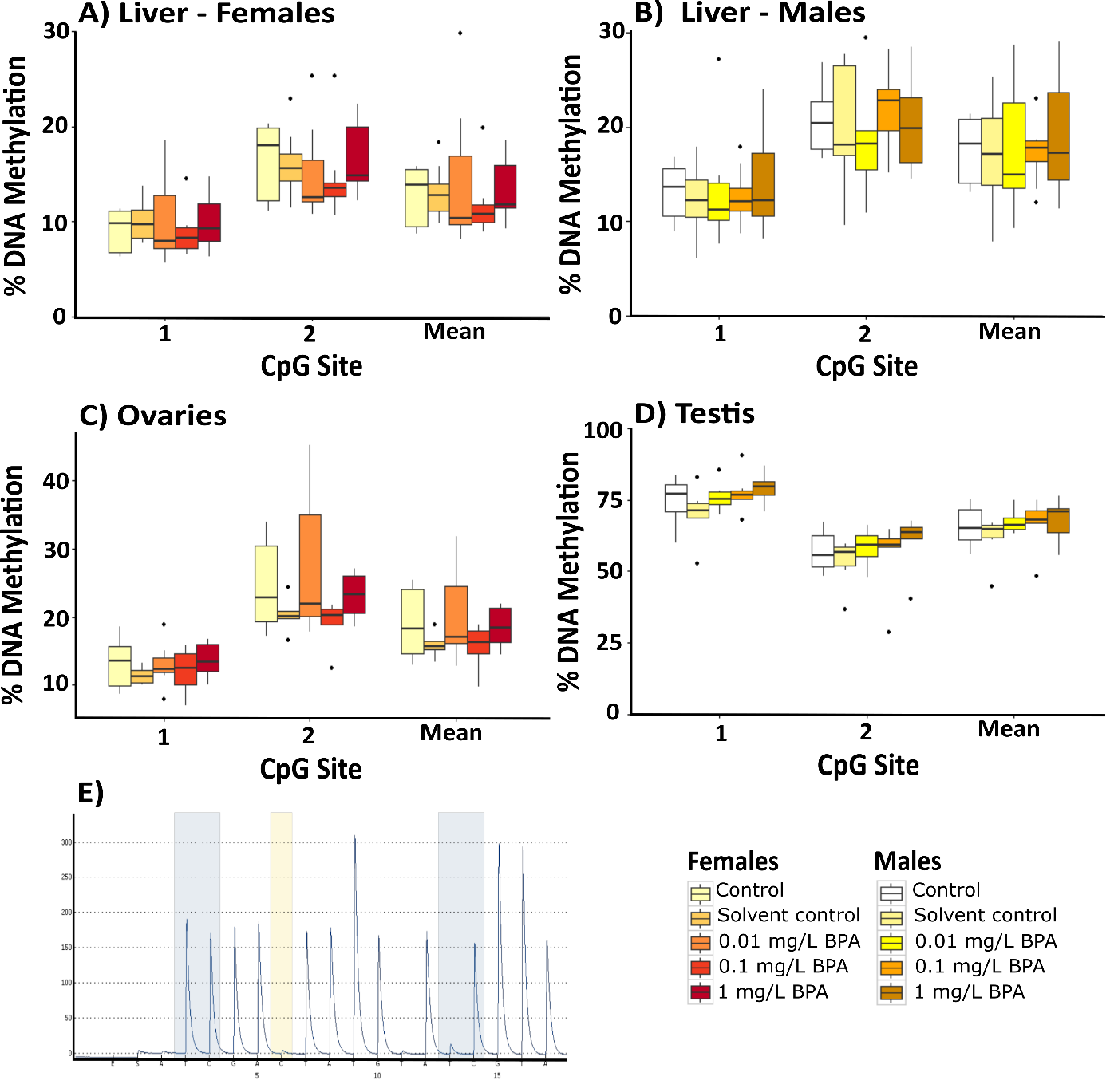

Supporting Information Figure S7.** Gene specific DNA methylation profiles for a series of two CpG sites in the promoter region of estrogen receptor 1 (*esr1*) in the liver of female **(A)** and male **(B)** adult zebrafish, and in the ovaries **(C)** and testes **(D)** of adult zebrafish following exposure to 0.01, 0.1 and 1 mg/L BPA. **E)** Example pyrogram of two CpG sites in the 5’ flanking regions of the *esr1* gene. Data are presented as boxplots (n = 6-8 for each group). Asterisks indicate significant differences compared to the solvent control (*P<0.05 **P<0.01 ***P<0.001).

**Supporting Information Table 1.** Measured concentrations of BPA in the exposure water, using HPLC-MS. Concentrations were measured for the three replicate treatment tanks on days 5, 10 and 15 of the exposure and are presented as mean values ± SEM.

| **Nominal  concentration** | **Control** | **Solvent control** | **0.01 mg/L BPA** | **0.1 mg/L BPA** | **1 mg/L BPA** |
| --- | --- | --- | --- | --- | --- |
| Day 5 | < 0.001 | < 0.001 | 0.02 ± 0.00 | 0.14 ± 0.01 | 1.28 ± 0.05 |
| Day 10 | < 0.001 | < 0.001 | 0.01 ± 0.00 | 0.14 ± 0.01 | 1.20 ± 0.14 |
| Day 15 | < 0.001 | < 0.001 | 0.01 ± 0.00 | 0.09 ± 0.03 | 1.43 ± 0.06 |
| **Mean** | **< 0.001** | **< 0.001** | **0.01** | **0.12** | **1.30** |

**Supporting Information Table 2.** Target genes, primer sequences and assay details for the RT-QPCR analysis.

| **Name** | **Symbol** | **Forward Primer (5’-3’)** | **Reverse Primer (5’-3’)** | **Product size (bp)** | **Ta (°C)** | **PCR efficiency** |
| --- | --- | --- | --- | --- | --- | --- |
| Ribosomal protein L8 | *rpl8* | CCGAGACCAAGAAATCCAGAG | CCAGCAACAACACCAACAAC | 91 | 59.5 | 1.95 |
| Aromatase | *cyp19a1a* | AGCCGTCCAGCCTCAG | ATCCAAAAGCAGAAGCAGTAG | 101 | 61.5 | 2.06 |
| Estrogen receptor 1 | *esr1* | TATGACCTGTTGCTGGAGATG | CGCCGTTGGACTGAATGG | 130 | 59.5 | 2.14 |
| Estrogen receptor 2a | *esr2a* | AGGAGAAAACCAAGTAAACCAATC | AGGCTGCTAACAAGGCTAATG | 173 | 59.0 | 1.86 |
| Estrogen receptor 2b | *esr2b* | ATCTGCTAATGCTGCTCTCAC | CGCTCTGTTGTCTGTCTTCC | 131 | 57.8 | 2.18 |
| Androgen receptor | *ar* | ACGAGGGTGTTAGATGAGAC | AAGTATGAGGAAAGCGAGTAAAG | 129 | 58.0 | 1.97 |
| Anti-Mullerian hormone | *amh* | TGTCTCAACCATCGTCTTCAG | CAGTCAATCCATCCATCCAAAC | 124 | 61.0 | 2.24 |
| Vitellogenin | *vtg1* | AGCAGCAGCAGTCGTAAC | CAATGATGGTGGCAGTCTTAG | 148 | 57.5 | 1.84 |
| DNA (cytosine-5)-methyltransferase 1 | *dnmt1* | CGCTGTCGTGTTGAGTATGC | TCCCTTGCCCTTTCCTTTCC | 180 | 58.5 | 2.06 |
| DNA (cytosine-5)-methyltransferase 3 | *dnmt3* | TGATGCCGTGAAAGTGAGTC | TTGCCGTGTAGTGATAGTGC | 172 | 58.5 | 2.19 |
| Histone deacetylase 1 | *hdac1* | TGACAAACGCATCTCCATTCG | CTCTTCTCCATCCTTCTCTTCTTC | 157 | 58.0 | 2.04 |
| Histone deacetylase 3 | *hdac3* | GAATGTGTGGAGTTTGTGAAGG | CTGGATGAAGTGTGAAGTCTGG | 190 | 57.0 | 1.98 |
| Methyl CpG binding protein 2 | *mecp2* | GAGGCAGAAACAGGACAG | TGGTGGTGATGATGATGG | 176 | 58.0 | 2.13 |
| Methyl-CpG-binding domain protein 2 | *mbd2* | AACAGCCTCCATCTTCAAG | CGTCCTCAGCACTTCTTC | 166 | 59.0 | 2.19 |
| Methyl-CpG-binding domain protein 3a | *mbd3a* | ACTCTTCTTTCGGCTCTG | TCTTCCTGCTTCCTGATG | 164 | 57.0 | 1.99 |

**Supporting Information Table 3.** Bisulfite-pyrosequencing primers and assay details for the gene promoters analyzed.

| **Name** | **Symbol** | **Forward Primer (5’-3’)** | **Reverse Primer (5’-3’)** | **Sequence Primer (5’-3’)** | **Sequence analysed (5’-3’)** | **Ta (°C)** |
| --- | --- | --- | --- | --- | --- | --- |
| Estrogen receptor 1 | *esr1* | AGAGGAGGTAAATAAATTAAAGATAGTTAG | Biotin-TACTCCTTTAACATATAATTTCCCATAACA | GGTAAATAAATTAAAGATAGTTAGG | TYGATATTGAYGGTTATTTTTTAGAGTAGGTTATGGTAATTAG | 58.0 |
| Anti-Mullerian hormone | *amh* | GTTTTTTATTTTTATGGGATGGTAGTTAGG | Biotin-AAACACAACTTAAAAACTTCCACTTATAT | TTGTTTTGAAGTATATTTGGAT | TATAYGTAATGGGGAATGTTTTAGTTTAAGGAAYGGTATTTGGTATTATTAAYGGGTTATTTATAAAATAATGTTTTTA | 58.0 |
| DNA (cytosine-5)-methyltransferase 1 | *dnmt1* | GGGTATTAATATGTGATAGTGTTAATTGTAG | Biotin - TAAACCCAAATACACTCACAACAC | TTATGAATTGTAGTTAGTAGTTGA | GAAATAYGYGYGGGTYGTTTTTTYGYGYGGAAAYGYGGGTGAGTYGGAYGTTATT | 58.0 |

**Supporting Information Table 4.** Statistical associations between **a)** BPA concentration and transcription; **b)** BPA concentration and global methylation; **c)** *dnmt1* transcription and global methylation; **d)** BPA concentration and specific CpG loci methylation; **e)** transcript expression and specific CpG loci methylation.

| **Table 4a. Regression analysis between BPA concentration and transcription .** | | | | |
| --- | --- | --- | --- | --- |
| **Tissue** | **Gene** | **-** | **Adjusted R2** | **P value** |
| Liver Female | *vtg1* | - | 0.155 | **0.018** |
|  | *esr1* | - | 0.142 | **0.049** |
|  | *esr2b* | - | -0.036 | 0.896 |
|  | *hdac1* | - | -0.045 | 0.718 |
| Liver Male | *vtg1* | - | 0.181 | **0.012** |
|  | *esr2a* | - | 0.021 | 0.246 |
|  | *esr2b* | - | 0.117 | 0.057 |
|  | *hdac1* | - | 0.141 | **0.033** |
| Ovary | *esr1* | - | 0.046 | 0.161 |
|  | *esr2a* | - | 0.238 | **0.017** |
|  | *esr2b* | - | 0.081 | 0.086 |
|  | *amh* | - | 0.021 | 0.248 |
|  | *cyp19a1a* | - | 0.031 | 0.220 |
|  | *ar* | - | 0.020 | 0.245 |
|  | *dnmt1* | - | -0.021 | 0.449 |
|  | *dnmt3* | - | 0.036 | 0.186 |
|  | *hdac1* | - | -0.035 | 0.674 |
|  | *hdac3* | - | 0.048 | 0.166 |
|  | *mecp2* | - | -0.043 | 0.751 |
|  | *mbd2* | - | -0.039 | 0.722 |
|  | *mbd3a* | - | 0.005 | 0.303 |
| Testis | *esr1* | - | -0.031 | 0.619 |
|  | *esr2a* | - | 0.053 | 0.121 |
|  | *esr2b* | - | 0.049 | 0.148 |
|  | *amh* | - | 0.075 | 0.094 |
|  | *cyp19a1a* | - | 0.189 | **0.025** |
|  | *ar* | - | -0.032 | 0.754 |
|  | *dnmt1* | - | 0.111 | **0.046** |
|  | *dnmt3* | - | 0.132 | **0.033** |
|  | *hdac1* | - | 0.059 | 0.117 |
|  | *hdac3* | - | 0.080 | 0.092 |
|  | *mecp2* | - | 0.083 | 0.072 |
|  | *mbd2* | - | 0.135 | **0.048** |
|  | *mbd3a* | - | 0.022 | 0.226 |
| **Table 4b. Regression analysis between BPA concentration and global methylation.** | | | | |
| **Tissue** | **Gene** | **-** | **Adjusted R2** | **P value** |
| Testis | - | **-** | 0.033 | 0.949 |
| Ovary | - | **-** | 0.051 | 0.121 |
| **Table 4c. Correlation analysis between *dnmt1* trancript expression and global methylation.** | | | | |
| **Tissue** | **Gene** | **-** | **Correlation coefficient** | **P value** |
| Testis | *dnmt1* | **-** | 0.110 | 0.576 |
| Ovary | *dnmt1* | **-** | 0.293 | 0.198 |
| **Table 4d. Regression analysis between BPA concentration and specific CpG loci methylation.** | | | | |
| **Tissue** | **Gene** | **CpG Position** | **Adjusted R2** | **P value** |
| Liver Female | *esr1* | 1 | -0.031 | 0.075 |
|  |  | 2 | -0.033 | 0.834 |
|  | *dnmt1* | 1 | 0.054 | 0.109 |
|  |  | 2 | 0.029 | 0.179 |
|  |  | 3 | 0.046 | 0.128 |
|  |  | 4 | 0.076 | 0.073 |
|  |  | 5 | 0.024 | 0.197 |
|  |  | 6 | 0.040 | 0.144 |
|  |  | 7 | 0.065 | 0.089 |
|  |  | 8 | 0.085 | 0.069 |
|  |  | 9 | 0.079 | 0.069 |
|  |  | 10 | 0.070 | 0.081 |
|  |  | 11 | 0.051 | 0.133 |
|  |  | Mean | 0.063 | 0.093 |
| Liver Male | *esr1* | 1 | -0.025 | 0.649 |
|  |  | 2 | -0.030 | 0.779 |
|  | *dnmt1* | 1 | -0.036 | 0.905 |
|  |  | 2 | -0.026 | 0.597 |
|  |  | 3 | -0.003 | 0.348 |
|  |  | 4 | -0.030 | 0.681 |
|  |  | 5 | -0.031 | 0.700 |
|  |  | 6 | -0.024 | 0.565 |
|  |  | 7 | -0.035 | 0.820 |
|  |  | 8 | -0.025 | 0.581 |
|  |  | 9 | -0.233 | 0.552 |
|  |  | 10 | -0.023 | 0.551 |
|  |  | 11 | -0.017 | 0.465 |
|  |  | Mean | -0.023 | 0.541 |
| Ovary | *esr1* | 1 | 0.052 | 0.115 |
|  |  | 2 | -0.024 | 0.583 |
|  | *amh* | 1 | 0.005 | 0.295 |
|  |  | 2 | 0.144 | 0.246 |
|  |  | 3 | -0.034 | 0.836 |
|  | *dnmt1* | 1 | 0.082 | 0.068 |
|  |  | 2 | 0.087 | 0.063 |
|  |  | 3 | 0.092 | 0.057 |
|  |  | 4 | 0.105 | **0.045** |
|  |  | 5 | 0.114 | **0.038** |
|  |  | 6 | 0.100 | **0.049** |
|  |  | 7 | 0.044 | 0.137 |
|  |  | 8 | 0.115 | **0.038** |
|  |  | 9 | 0.091 | 0.058 |
|  |  | 10 | 0.098 | 0.051 |
|  |  | 11 | 0.061 | 0.100 |
|  |  | Mean | 0.094 | 0.055 |
| Testis | *esr1* | 1 | -0.016 | 0.465 |
|  |  | 2 | -0.009 | 0.397 |
|  | *amh* | 1 | 0.163 | **0.013** |
|  |  | 2 | 0.036 | 0.152 |
|  |  | 3 | -0.017 | 0.497 |
|  | *dnmt1* | 1 | -0.380 | **0.047** |
|  |  | 2 | 0.003 | 0.304 |
|  |  | 3 | 0.011 | 0.255 |
|  |  | 4 | -0.016 | 0.480 |
|  |  | 5 | 0.000 | 0.318 |
|  |  | 6 | 0.000 | 0.325 |
|  |  | 7 | -0.016 | 0.471 |
|  |  | 8 | -0.001 | 0.334 |
|  |  | 9 | 0.006 | 0.290 |
|  |  | 10 | 0.038 | 0.182 |
|  |  | 11 | 0.005 | 0.313 |
|  |  | Mean | -0.001 | 0.335 |
| **Table 4e. Correlation analysis between trancript expression and specific CpG loci methylation.** | | | | |
| **Tissue** | **Gene** | **CpG Position** | **Correlation coefficient** | **P value** |
| Ovary | *esr1* | 1 | -0.229 | 0.281 |
|  |  | 2 | -0.225 | 0.289 |
|  | *amh* | 1 | -0.323 | 0.164 |
|  |  | 2 | -0.286 | 0.235 |
|  |  | 3 | -0.286 | 0.221 |
|  | *dnmt1* | 1 | -0.050 | 0.830 |
|  |  | 2 | -0.026 | 0.912 |
|  |  | 3 | -0.003 | 0.991 |
|  |  | 4 | -0.142 | 0.540 |
|  |  | 5 | 0.097 | 0.674 |
|  |  | 6 | 0.082 | 0.724 |
|  |  | 7 | 0.192 | 0.404 |
|  |  | 8 | 0.065 | 0.780 |
|  |  | 9 | -0.033 | 0.887 |
|  |  | 10 | -0.055 | 0.814 |
|  |  | 11 | -0.068 | 0.771 |
|  |  | Mean | 0.023 | 0.921 |
| Testis | *esr1* | 1 | 0.095 | 0.653 |
|  |  | 2 | 0.386 | **0.035** |
|  | *amh* | 1 | -0.452 | **0.014** |
|  |  | 2 | -0.047 | 0.815 |
|  |  | 3 | -0.214 | 0.285 |
|  | *dnmt1* | 1 | -0.024 | 0.903 |
|  |  | 2 | -0.180 | 0.359 |
|  |  | 3 | -0.204 | 0.306 |
|  |  | 4 | -0.157 | 0.425 |
|  |  | 5 | -0.523 | **0.004** |
|  |  | 6 | -0.514 | **0.006** |
|  |  | 7 | -0.475 | **0.014** |
|  |  | 8 | -0.435 | **0.023** |
|  |  | 9 | -0.382 | 0.066 |
|  |  | 10 | -0.035 | 0.886 |
|  |  | 11 | -0.039 | 0.889 |
|  |  | Mean | -0.380 | **0.047** |
